# Characteristics of Outreach patients that received end-of-life counseling

**DOI:** 10.1186/cc9938

**Published:** 2011-03-11

**Authors:** K Kyeremanteng, RP Patel, EF Fitzgibbon

**Affiliations:** 1 The Ottawa Hospital, Ottawa, Canada

## Introduction

Approximately 20 to 30% of ICU patients are palliated in the ICU. Many of these patients have not had goals of care discussions prior to being admitted to the ICU. Several of these patients may have prolonged courses that can cause anguish for patients and their families and may have been prevented if goals of care discussions occurred earlier. There has been increasing evidence that palliative care involvement in critical care improves outcomes such as quality of end-of-life care [1], decreased length of stay [2] and better pain and symptom management [3]. No studies have looked at medical emergency teams/Outreach with respect to palliative care and end-of-life care. We performed a retrospective descriptive study looking at the characteristics of Outreach patients who received end-of-life counseling (EOLC).

## Methods

We evaluated 80 patients from The Ottawa Hospital General campus that were seen by Outreach and received EOLC in 2007. From the Outreach database and the hospital computerized health record system, we obtained patient demographics and medical information such as admission diagnosis and reason for Outreach call. We compared these patients with ones that did not receive EOLC. We also subdivided the patients that received EOLC into patients that were successfully palliated versus ones that were not palliated and compared patient characteristics.

## Results

Twenty-one percent of all Outreach patients received EOLC in 2007. Comparing patients that received EOLC with those with no EOLC, mean age was 72.3 ± 11.5 versus 68.9 ± 17.6 (mean ± SD). Fifty-two percent had cancer versus 38%. Dementia was involved in 17% of EOLC patients versus 8% in non-EOLC patients. Length of stay (LOS) was 26.3 ± 26.1 days versus 34 ± 30.7. Admission to Oncology/Hematology/Radiation Oncology was 33% in the EOLC group compared with 20%. The proportion of patients seen during the day was 49% versus 64%. Call indication was mostly respiratory in the EOLC group (53% vs. 32%). Sex, number of co-morbidities, days admitted prior to Outreach call and admission diagnosis were similar in both groups. Amongst the patients that received EOLC, 49% were palliated and 51% were not palliated. Patient characteristics were similar in these two groups. (*t*-Score testing is pending.)

## Conclusions

At our tertiary center, the Outreach patients that receive EOLC tend to be older, admitted for respiratory illness and have a diagnosis of cancer.
